# Supplementary material for: Divergent Asymmetric Total Synthesis of All Four Pestalotin Diastereomers from (R)-Glycidol
Source: Molecules. 2020 Jan 17;25(2):394. doi: 10.3390/molecules25020394 (PMC7024299; doi:10.3390/molecules25020394)
Supplement: Supplementary file 1 [file molecules-25-00394-s001.zip › Molecules-Pestalotin-NMR.pdf]

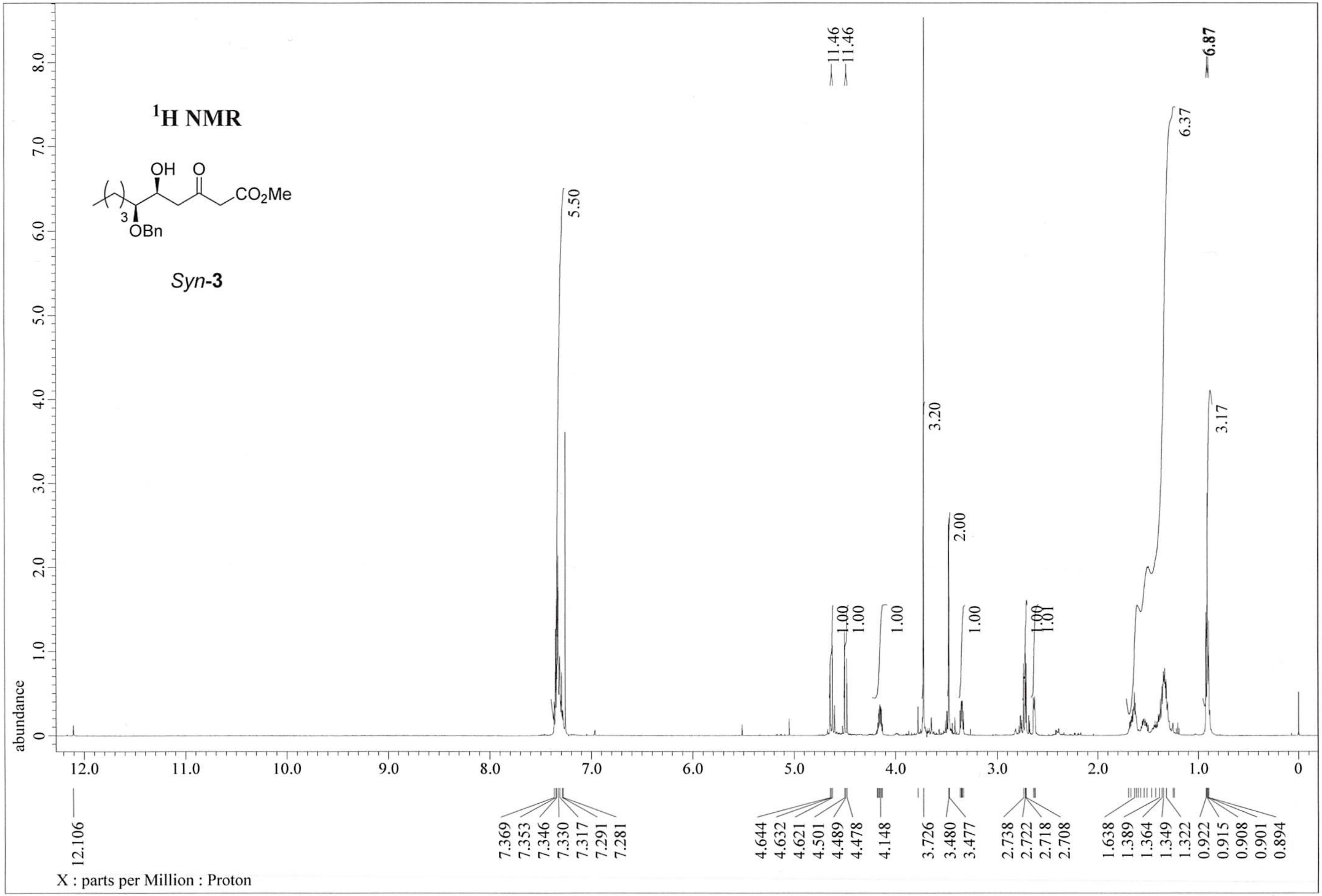
1H NMR
OH
O
CO2Me
3
OBn
Syn-3
abundance
X : parts per Million : Proton
12.106
7.369
7.353
7.346
7.330
7.317
7.291
7.281
4.644
4.632
4.621
4.501
4.489
4.478
4.148
3.726
3.480
3.477
2.738
2.722
2.718
2.708
1.638
1.389
1.364
1.349
1.322
0.922
0.915
0.908
0.901
0.894
5.50
1.00
1.00
1.00
3.20
2.00
1.00
1.00
1.01
6.37
3.17
11.46
11.46
6.87

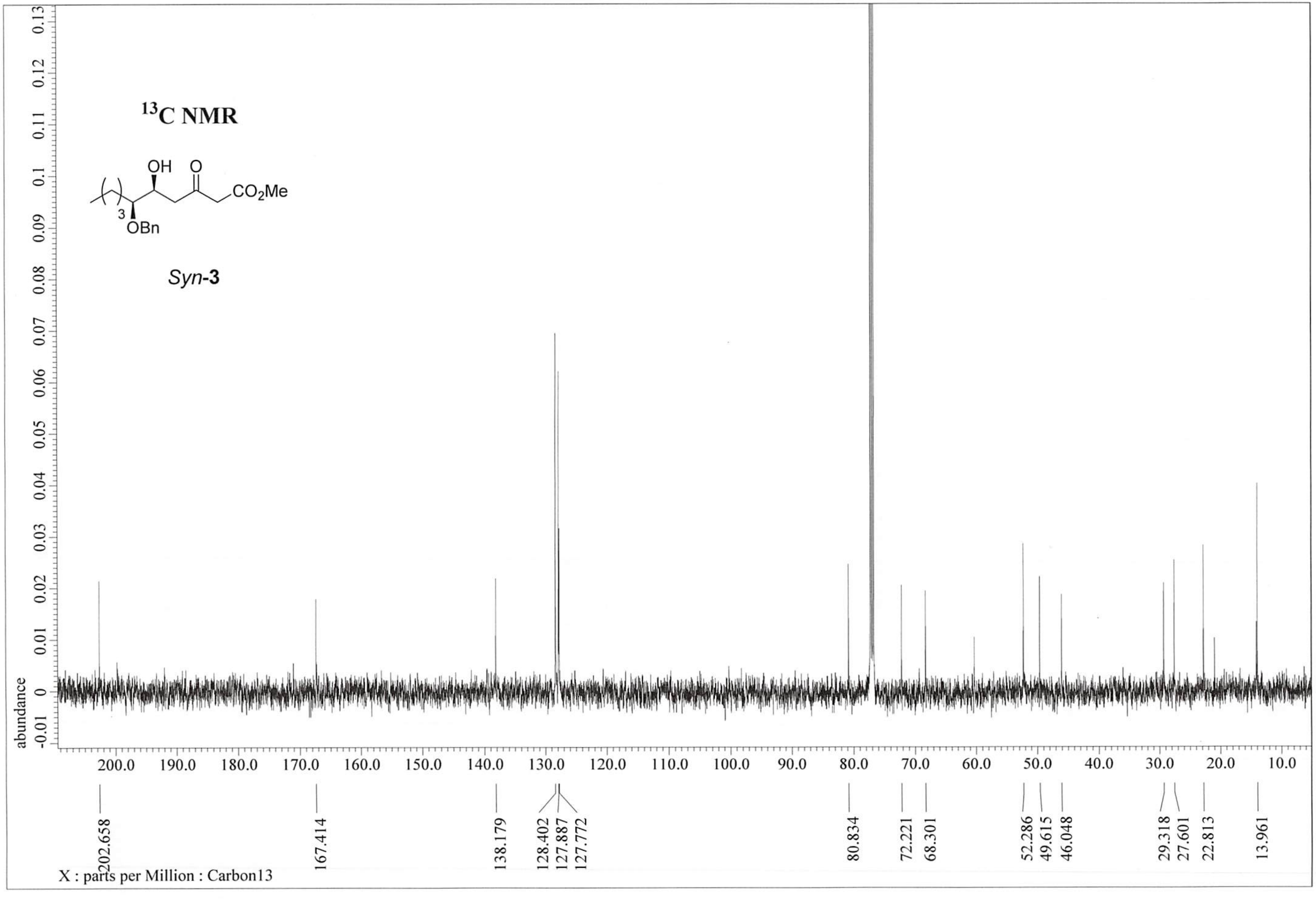

13C NMR
OH
O
CO2Me
3
OBn
Syn-3
abundance
202.658
167.414
138.179
128.402
127.887
127.772
80.834
72.221
68.301
52.286
49.615
46.048
29.318
27.601
22.813
13.961
X : parts per Million : Carbon13

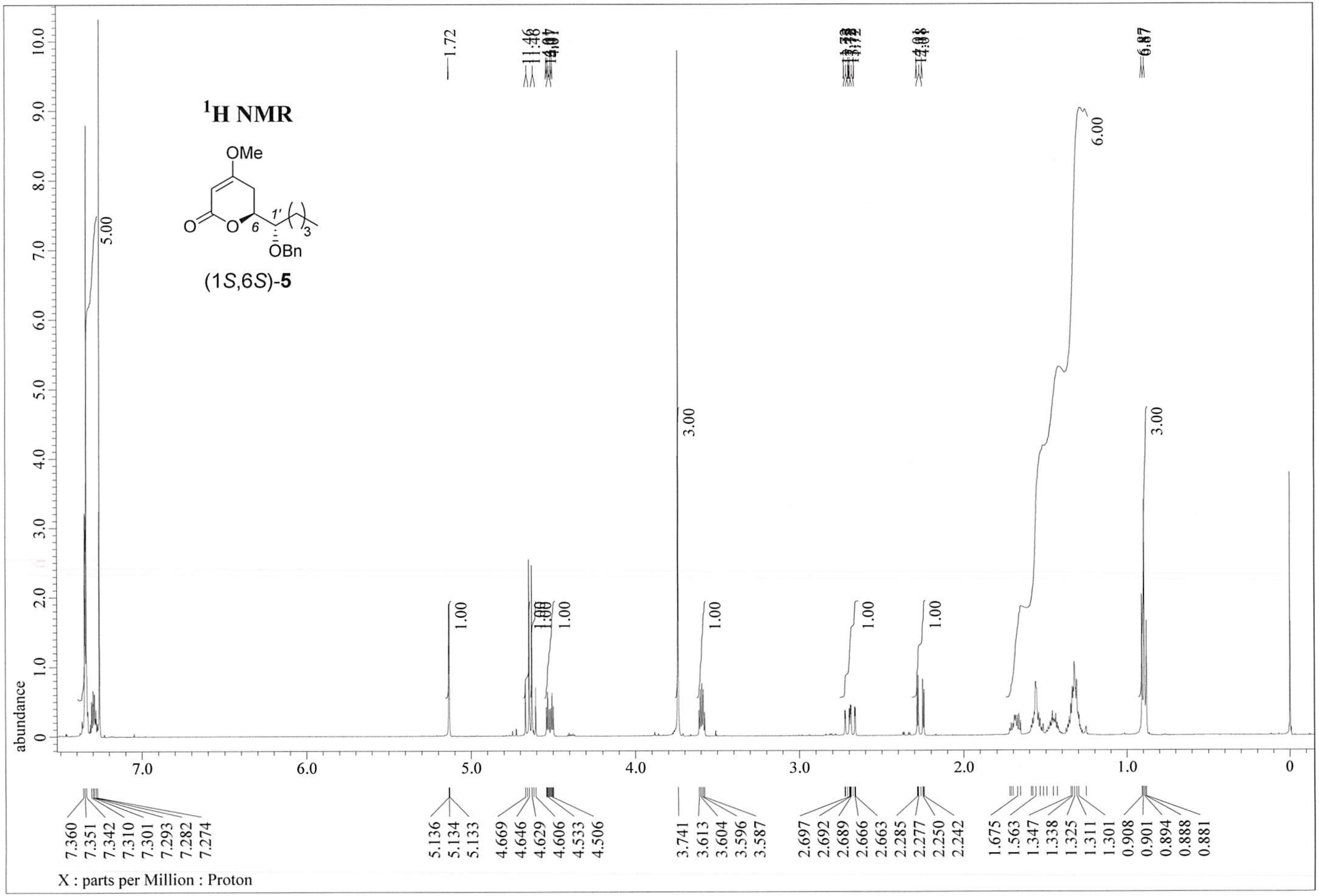

¹H NMR
OMe
O
O
6
1'
3
OBn
(1S,6S)-5
abundance
X : parts per Million : Proton
7.360
7.351
7.342
7.310
7.301
7.293
7.282
7.274
5.136
5.134
5.133
4.669
4.646
4.629
4.606
4.533
4.506
3.741
3.613
3.604
3.596
3.587
2.697
2.692
2.689
2.666
2.663
2.285
2.277
2.250
2.242
1.675
1.563
1.347
1.338
1.325
1.311
1.301
0.908
0.901
0.894
0.888
0.881
5.00
1.00
1.00
1.00
3.00
1.00
1.00
1.00
6.00
3.00
1.72
11.46

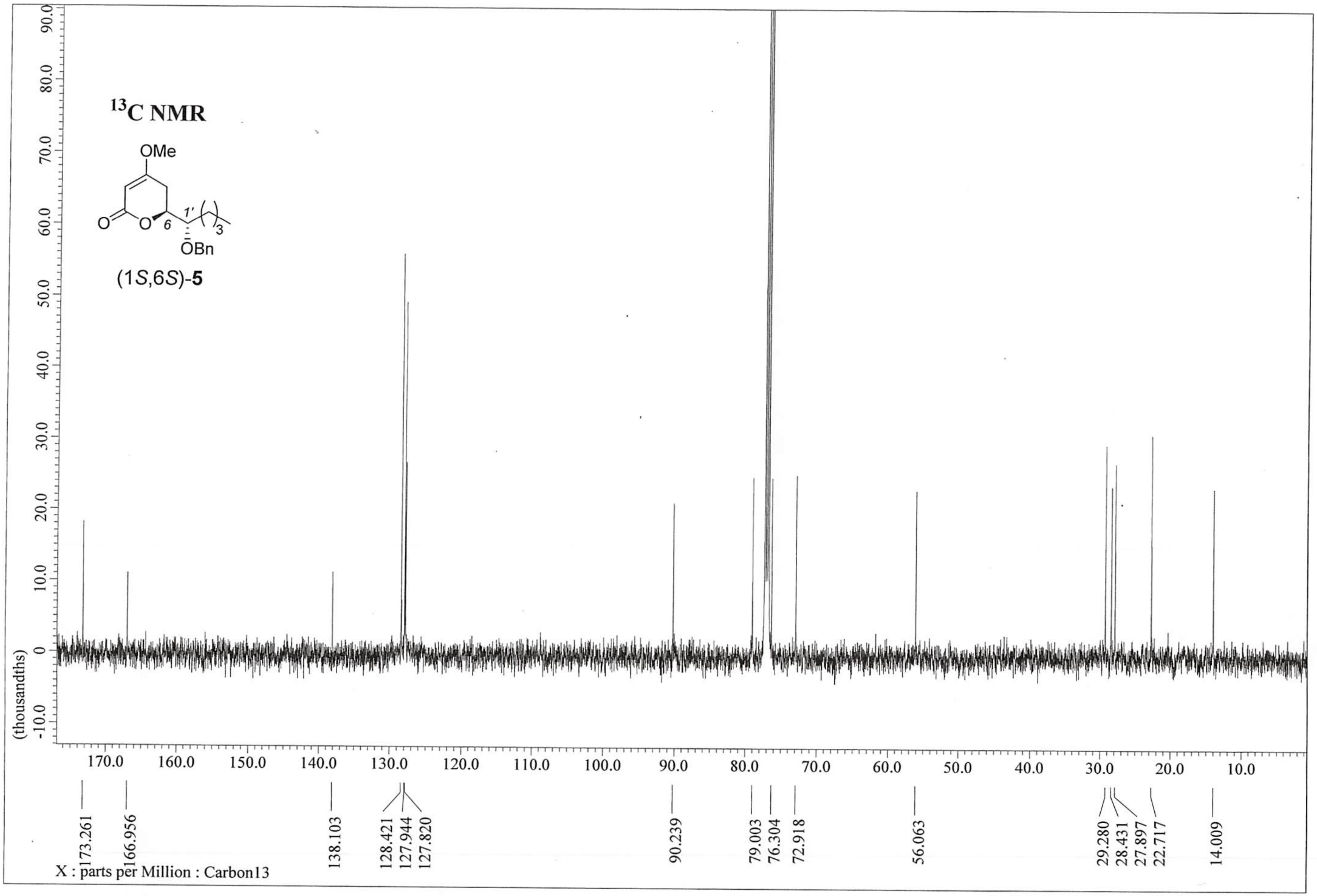

13C NMR
OMe
O
O
6
1'
3
OBn
(1S,6S)-5
(thousandths)
173.261
166.956
138.103
128.421
127.944
127.820
90.239
79.003
76.304
72.918
56.063
29.280
28.431
27.897
22.717
14.009
X : parts per Million : Carbon13

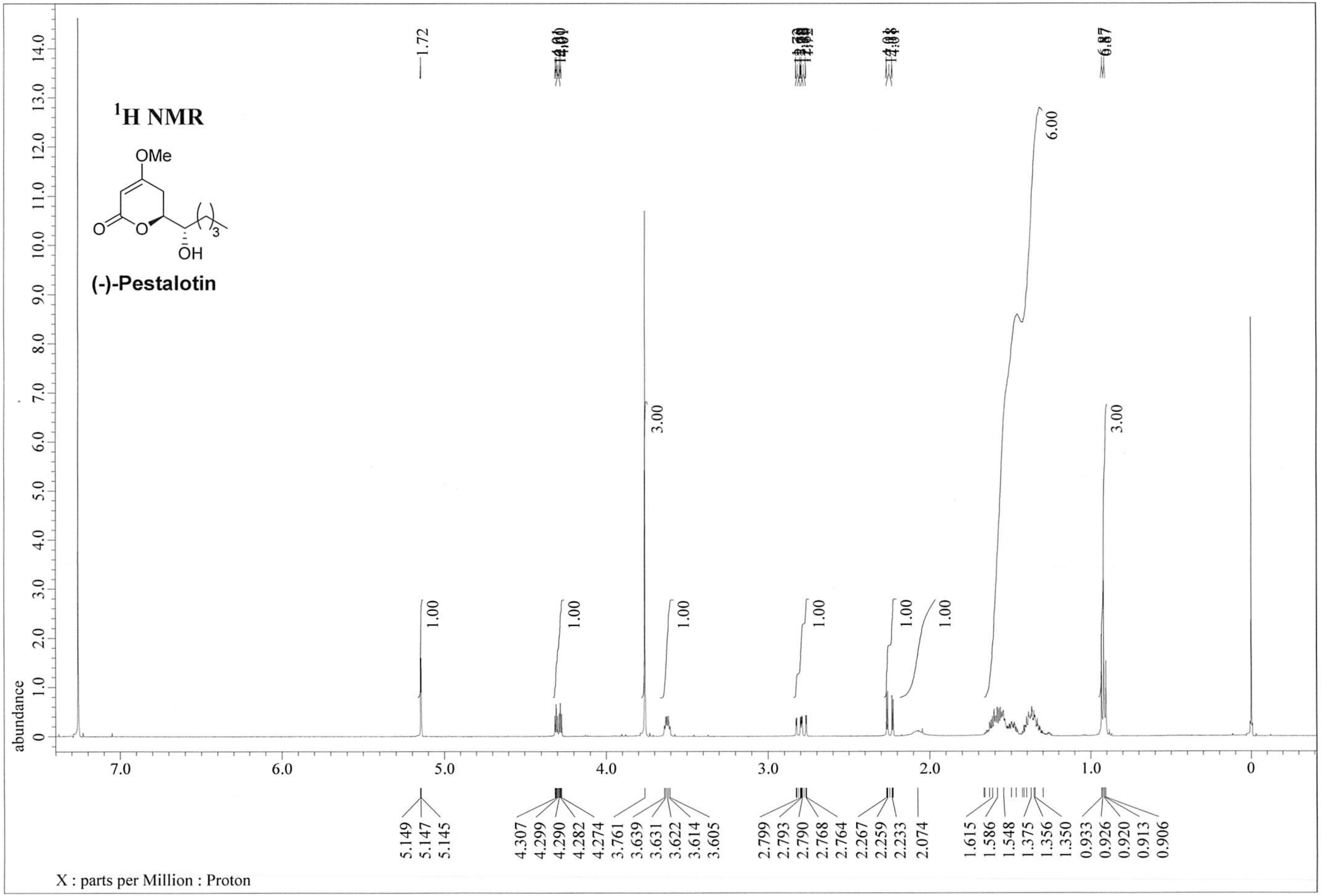

1H NMR
OMe
OH
3
(-)-Pestalotin
abundance
X : parts per Million : Proton
1.72
6.87
1.00
3.00
6.00
5.149
5.147
5.145
4.307
4.299
4.290
4.282
4.274
3.761
3.639
3.631
3.622
3.614
3.605
2.799
2.793
2.790
2.768
2.764
2.267
2.259
2.233
2.074
1.615
1.586
1.548
1.375
1.356
1.350
0.933
0.926
0.920
0.913
0.906

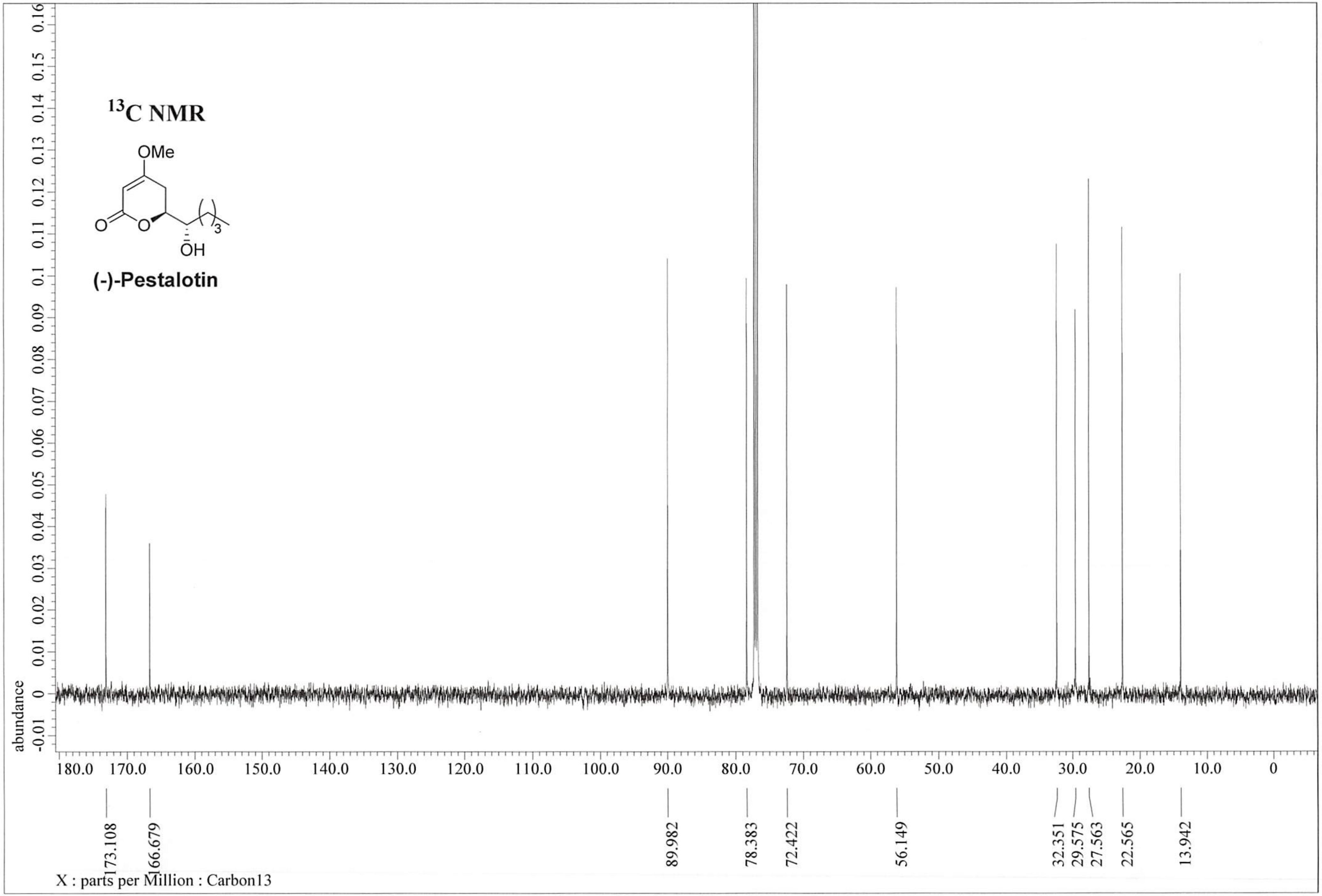

13C NMR
OMe
O
O
OH
3
(-)-Pestalotin
abundance
-0.01
0
0.01
0.02
0.03
0.04
0.05
0.06
0.07
0.08
0.09
0.1
0.11
0.12
0.13
0.14
0.15
0.16
180.0
170.0
160.0
150.0
140.0
130.0
120.0
110.0
100.0
90.0
80.0
70.0
60.0
50.0
40.0
30.0
20.0
10.0
0
173.108
166.679
89.982
78.383
72.422
56.149
32.351
29.575
27.563
22.565
13.942
X : parts per Million : Carbon13

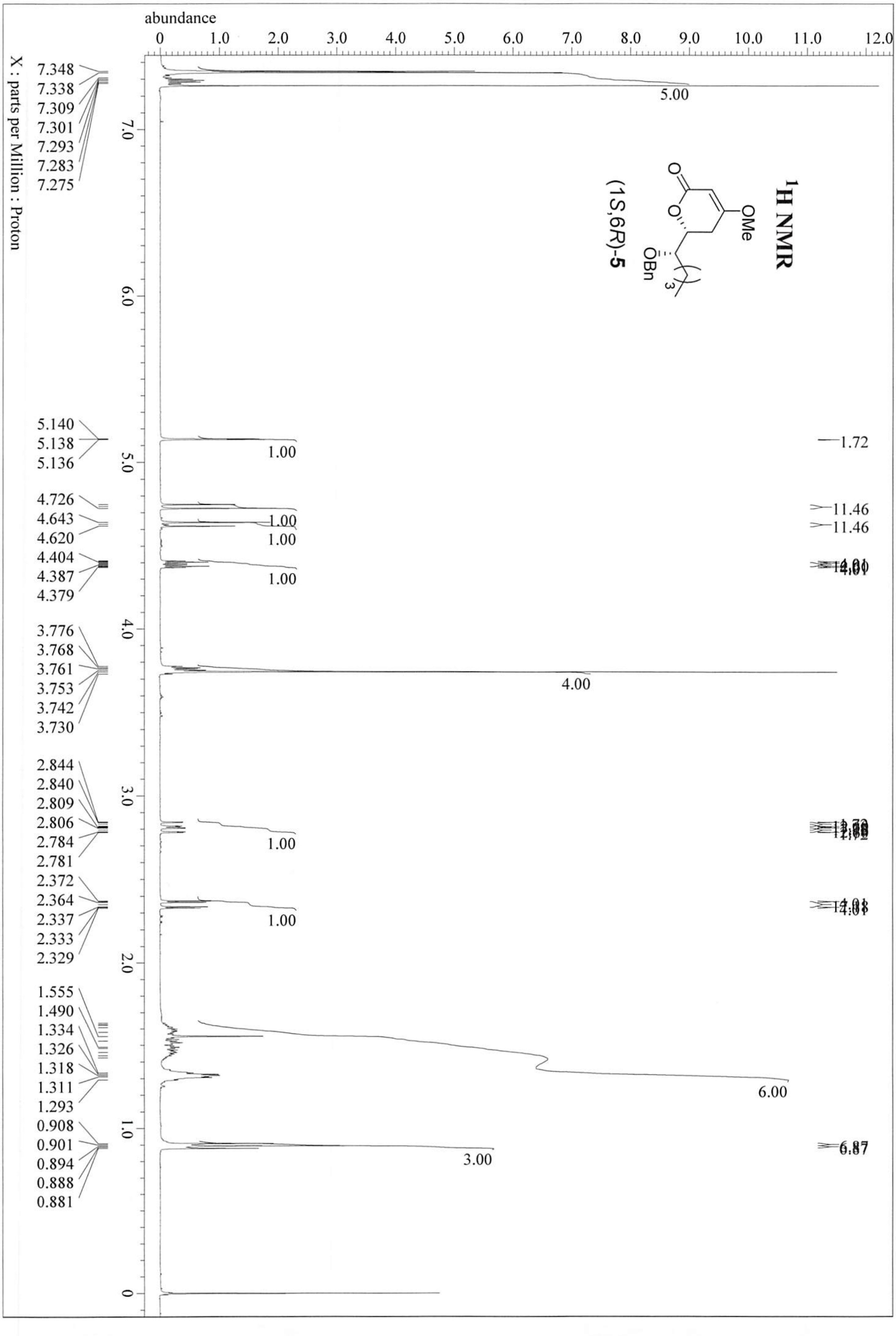

abundance
0
1.0
2.0
3.0
4.0
5.0
6.0
7.0
8.0
9.0
10.0
11.0
12.0
X : parts per Million : Proton
7.348
7.338
7.309
7.301
7.293
7.283
7.275
5.140
5.138
5.136
4.726
4.643
4.620
4.404
4.387
4.379
3.776
3.768
3.761
3.753
3.742
3.730
2.844
2.840
2.809
2.806
2.784
2.781
2.372
2.364
2.337
2.333
2.329
1.555
1.490
1.334
1.326
1.318
1.311
1.293
0.908
0.901
0.894
0.888
0.881
5.00
1.00
1.00
1.00
1.00
4.00
1.00
1.00
6.00
3.00
1.72
11.46
11.46
6.87
^{1}H NMR
OMe
OBn
(1S,6R)-5

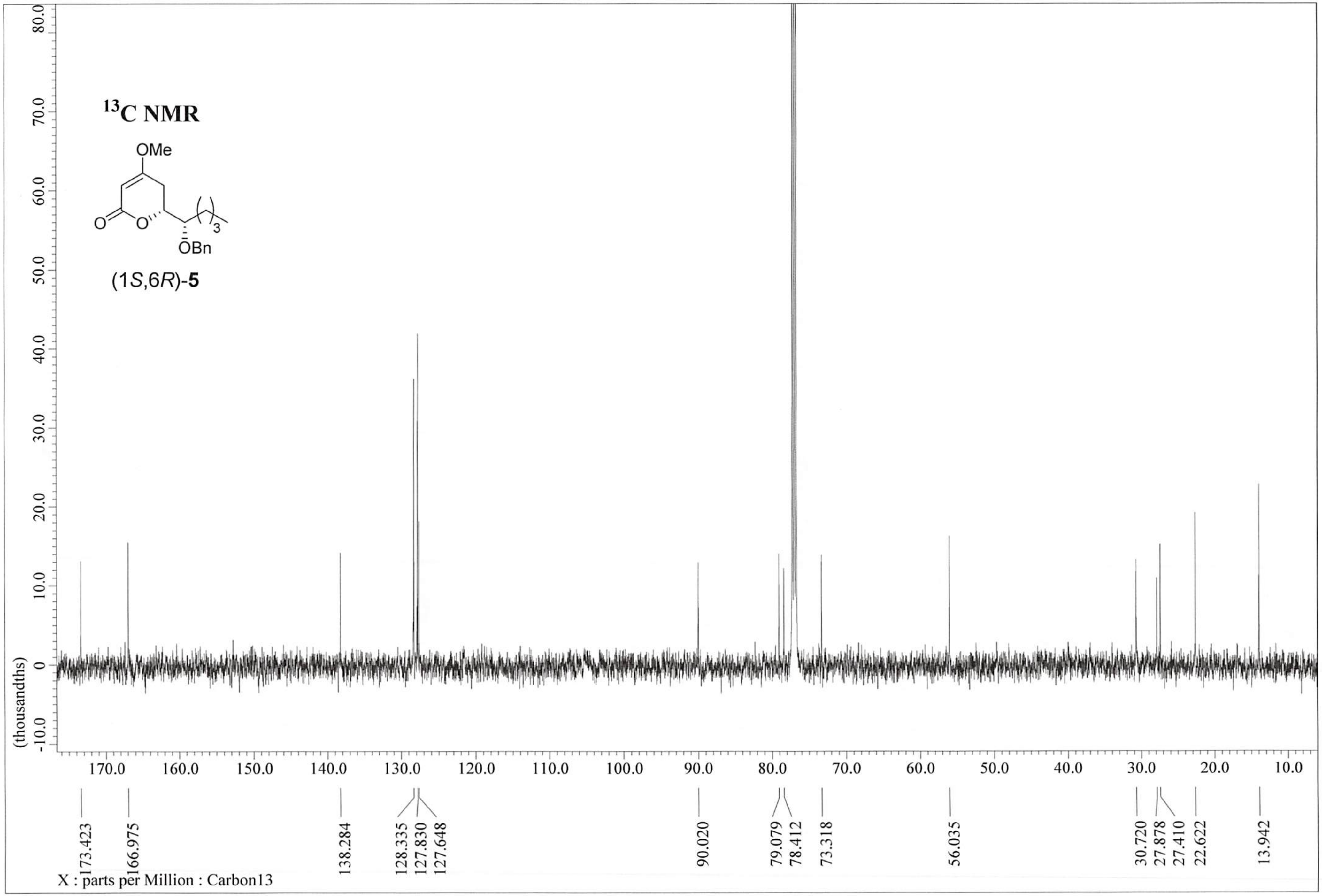

13C NMR
OMe
O
O
OBn
3
(1S,6R)-5
(thousandths)
173.423
166.975
138.284
128.335
127.830
127.648
90.020
79.079
78.412
73.318
56.035
30.720
27.878
27.410
22.622
13.942
X : parts per Million : Carbon13

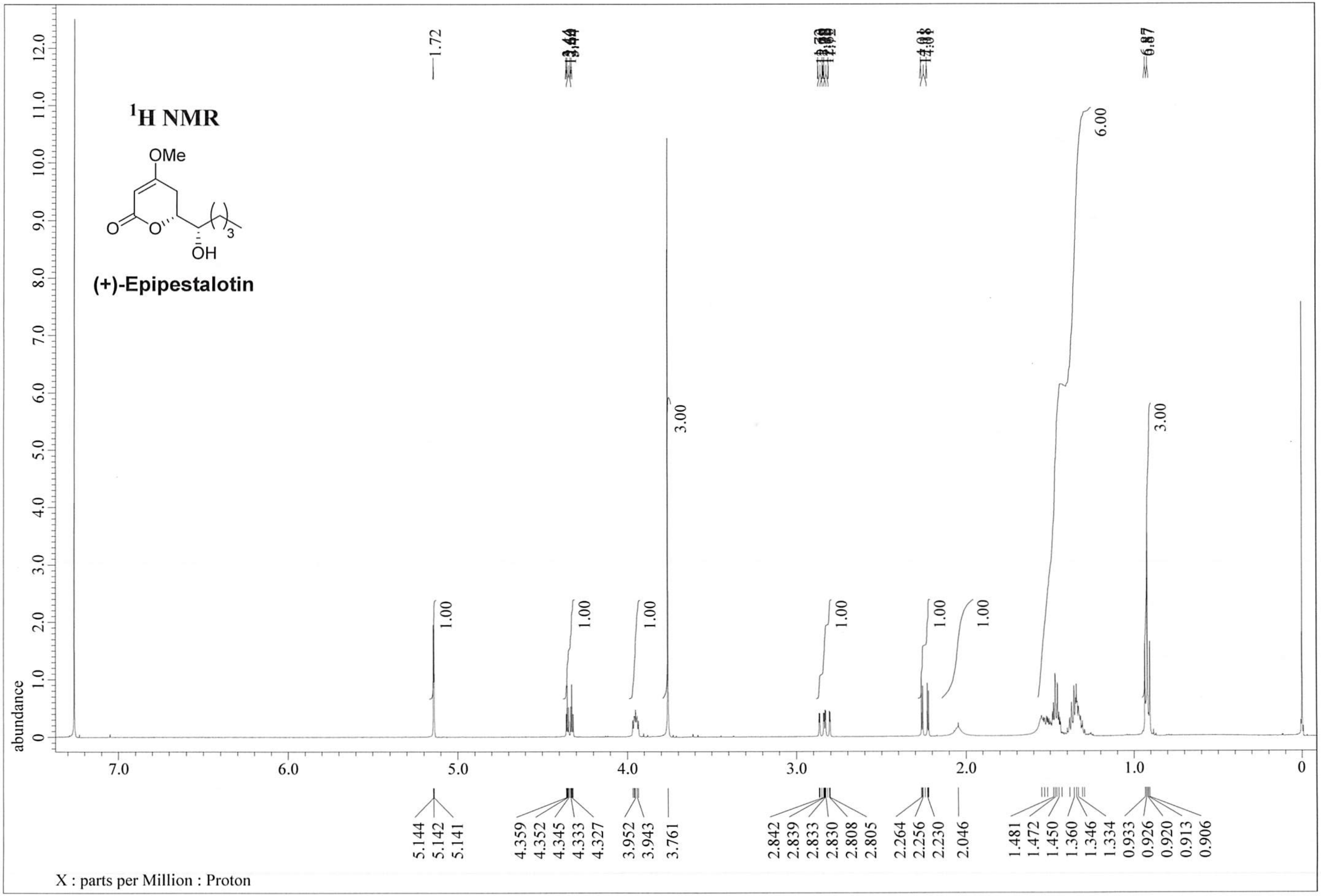

1H NMR
OMe
O
O
3
OH
(+)-Epipestalotin
abundance
X : parts per Million : Proton
5.144
5.142
5.141
4.359
4.352
4.345
4.333
4.327
3.952
3.943
3.761
2.842
2.839
2.833
2.830
2.808
2.805
2.264
2.256
2.230
2.046
1.481
1.472
1.450
1.360
1.346
1.334
0.933
0.926
0.920
0.913
0.906
1.00
1.00
1.00
3.00
1.00
1.00
1.00
6.00
3.00

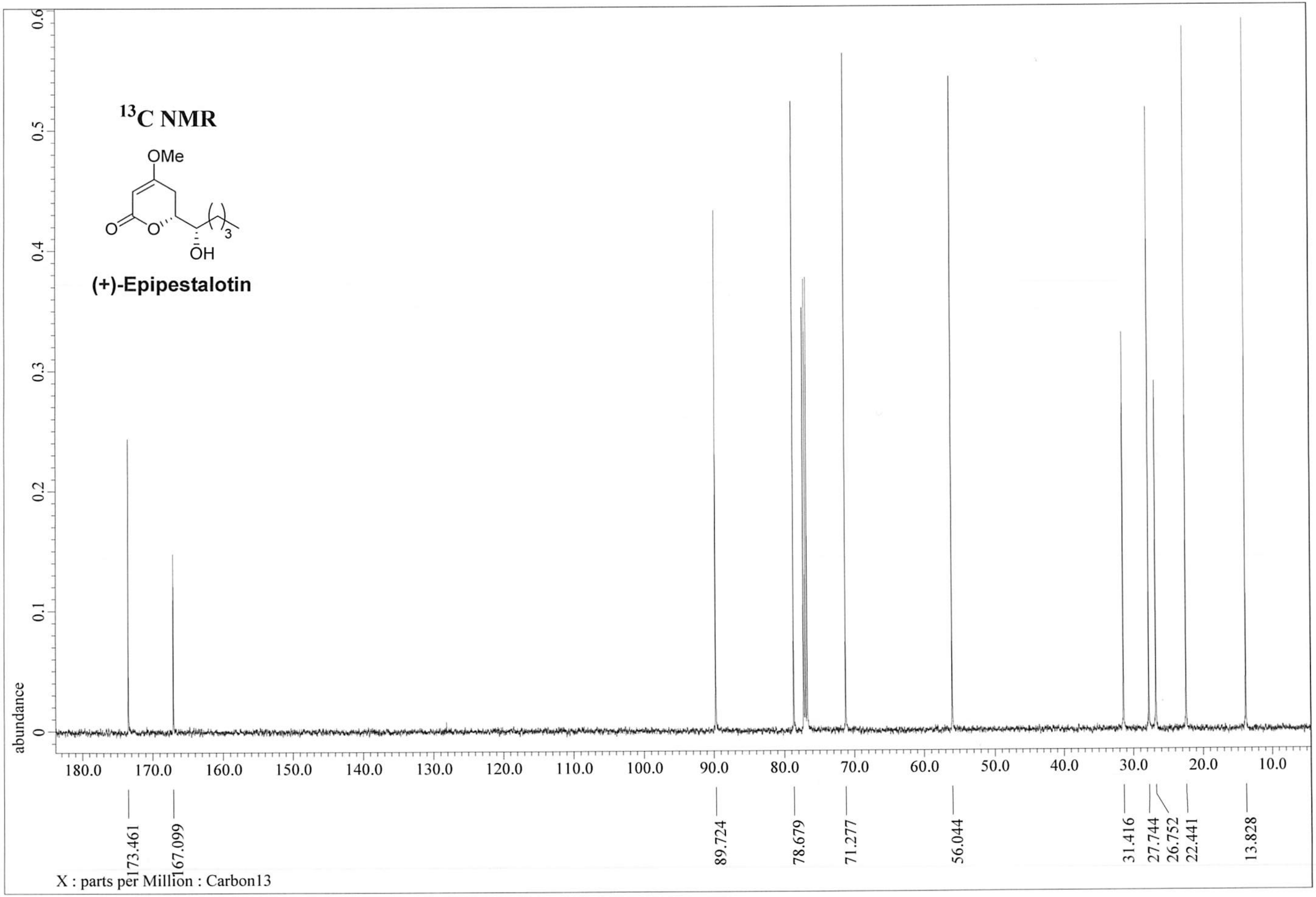

13C NMR
OMe
O
O
OH
3
(+)-Epipestalotin
abundance
0
0.1
0.2
0.3
0.4
0.5
0.6
180.0
170.0
160.0
150.0
140.0
130.0
120.0
110.0
100.0
90.0
80.0
70.0
60.0
50.0
40.0
30.0
20.0
10.0
173.461
167.099
89.724
78.679
71.277
56.044
31.416
27.744
26.752
22.441
13.828
X : parts per Million : Carbon13
