# Supplementary figures and images for: Divergent Asymmetric Total Synthesis of All Four Pestalotin Diastereomers from (R)-Glycidol
Source: Molecules. 2020 Jan 17;25(2):394. doi: 10.3390/molecules25020394 (PMC7024299; doi:10.3390/molecules25020394)

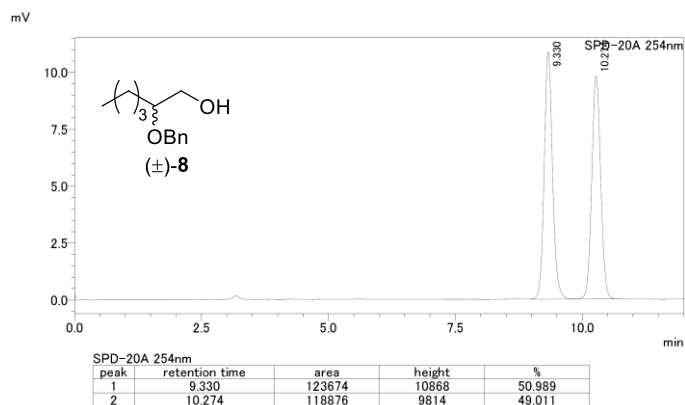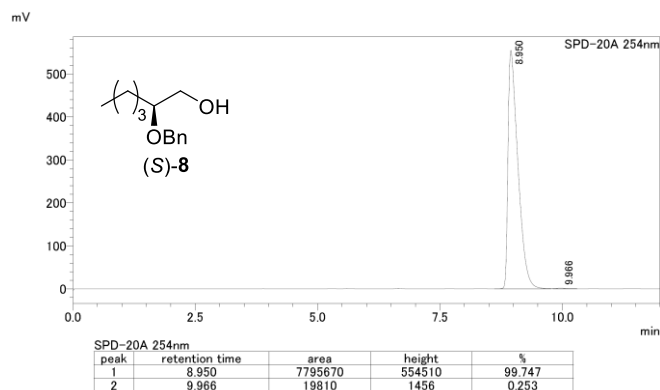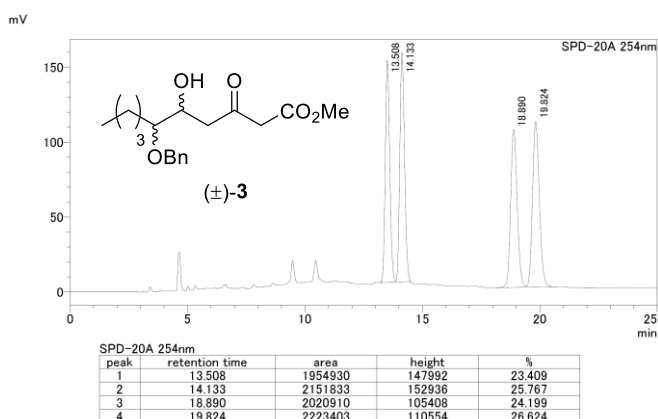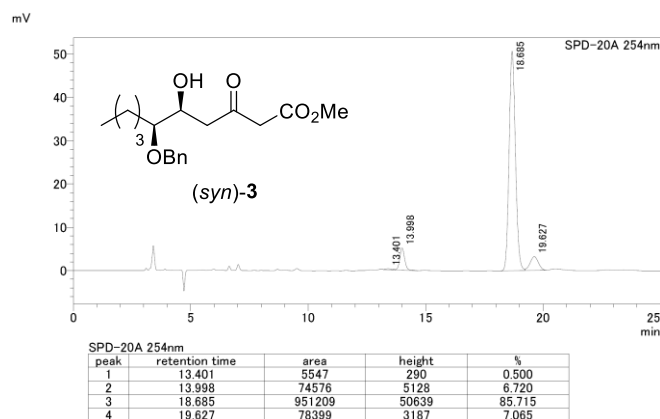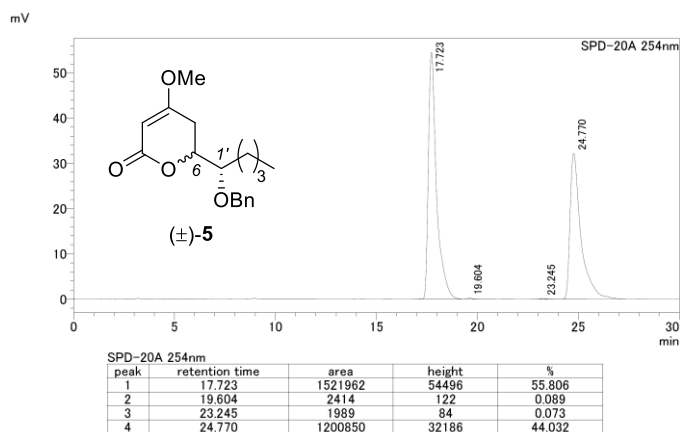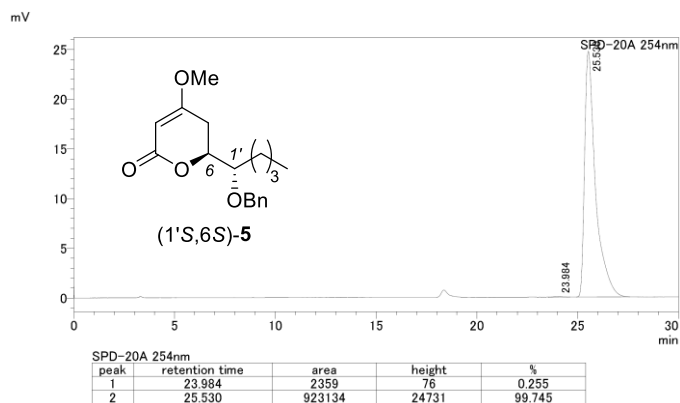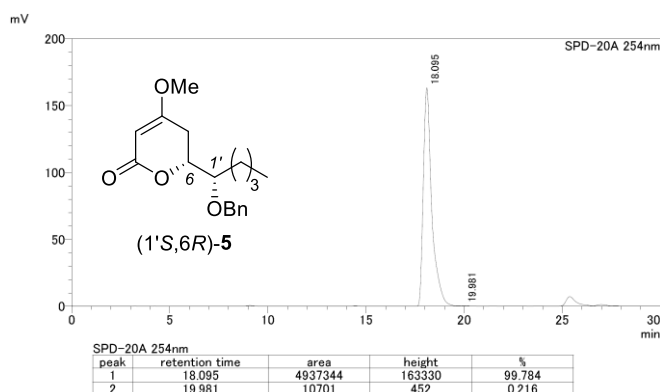

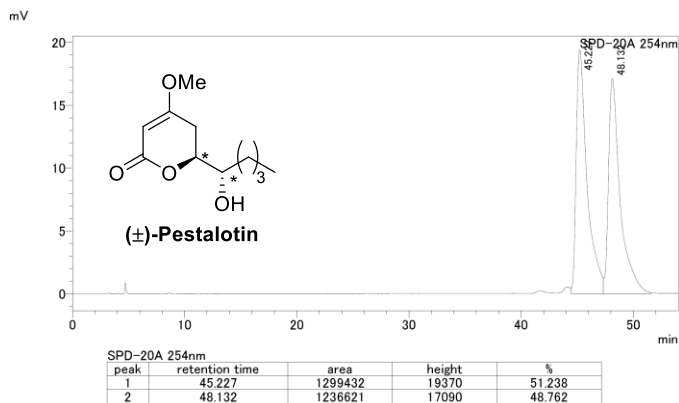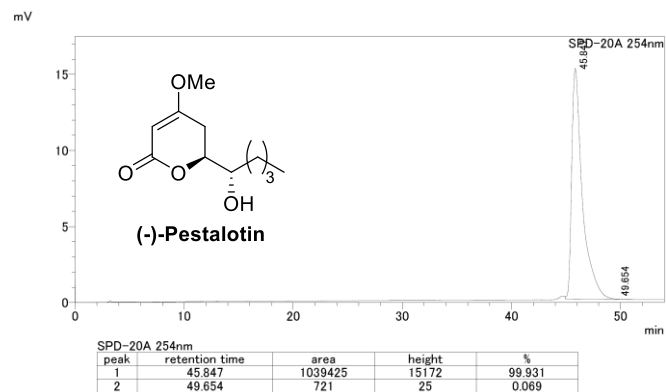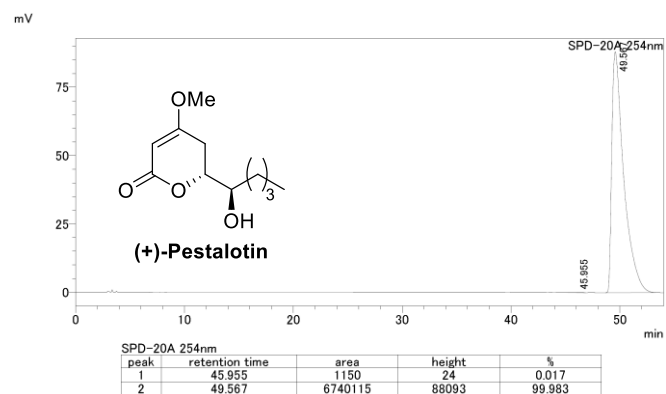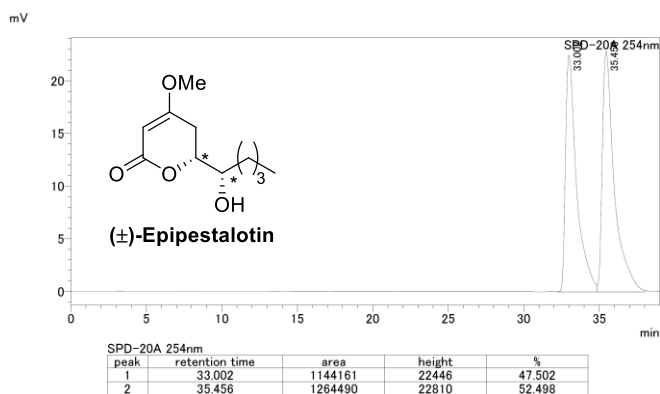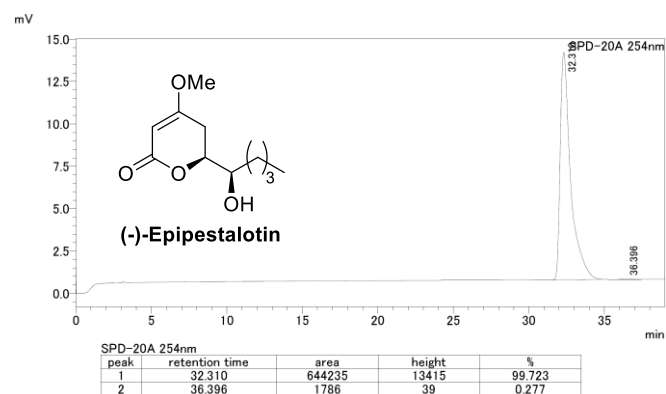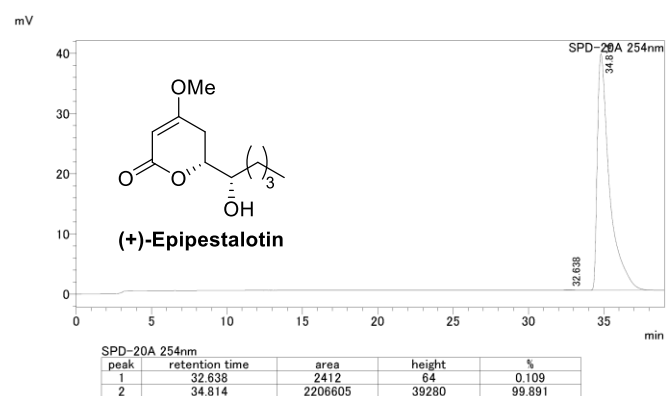

Supplement: Supplementary file 1 [file molecules-25-00394-s001.zip › Molecules-Pestalotin-HPLC.pdf]
